# Supplementary figures and images for: Temporal assessment of entomological surveillance of Trypanosoma cruzi vectors in an endemic area of northeastern Brazil
Source: PLoS One. 2023 Jun 15;18(6):e0287260. doi: 10.1371/journal.pone.0287260 (PMC10270571; doi:10.1371/journal.pone.0287260)

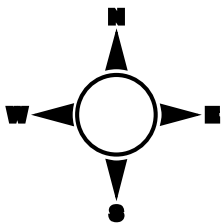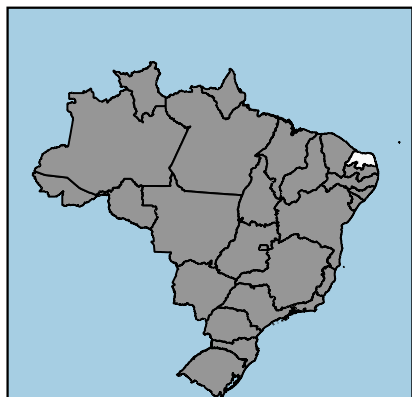

0 1,000 km

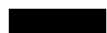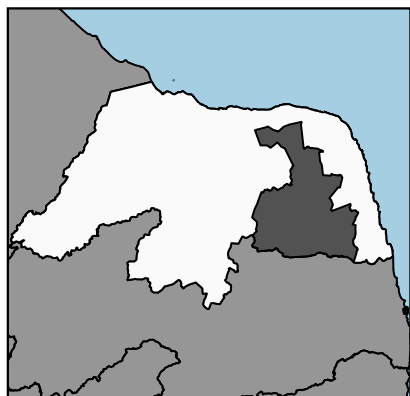

0 100 km

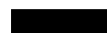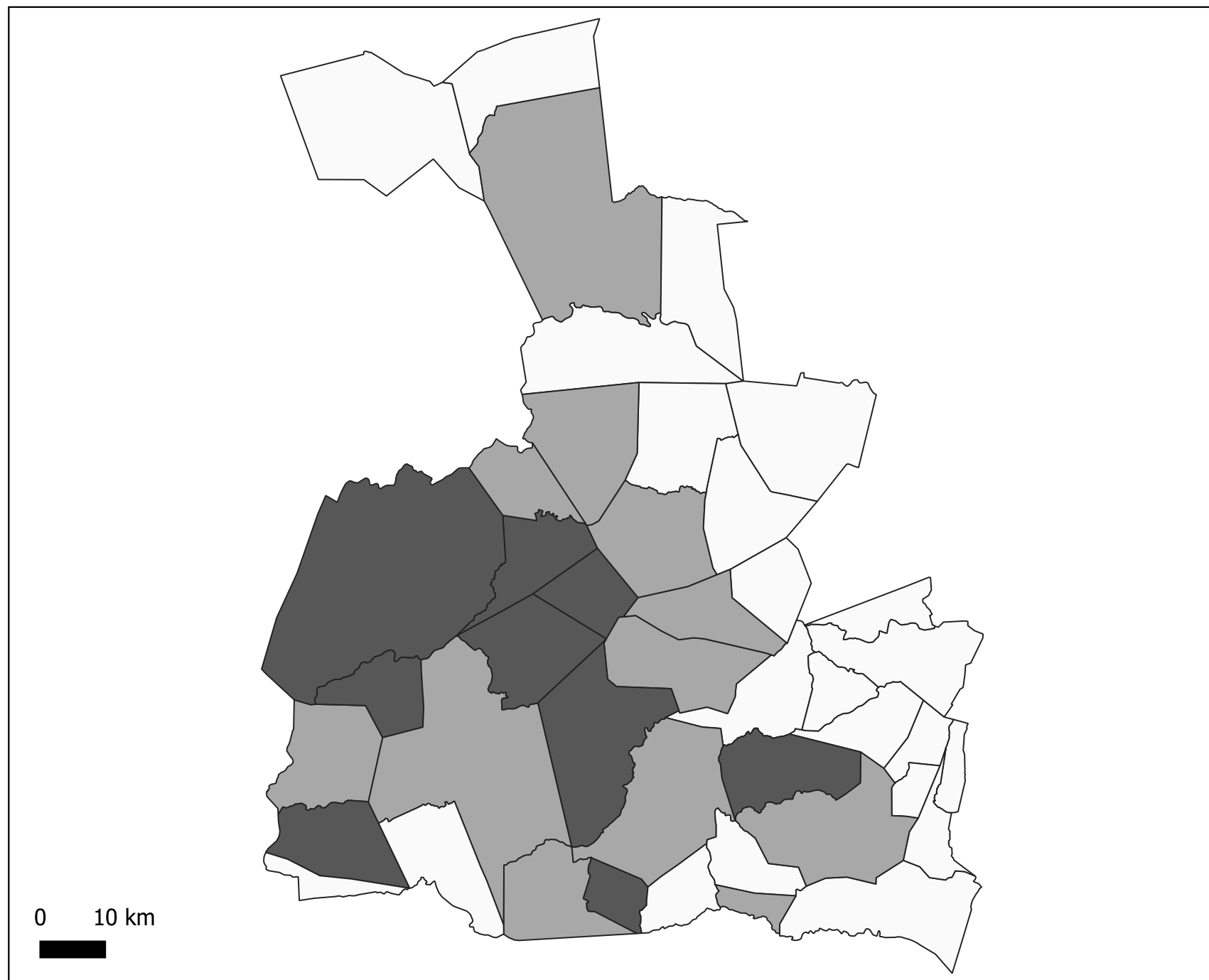

0 10 km

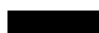

Risk of vectoral transmission

Low Medium High

Supplement: S1 File — (PDF) [file pone.0287260.s001.pdf]

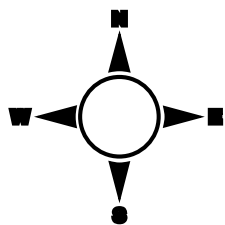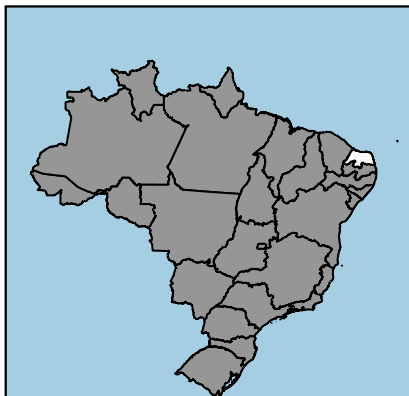

0 1,000 km

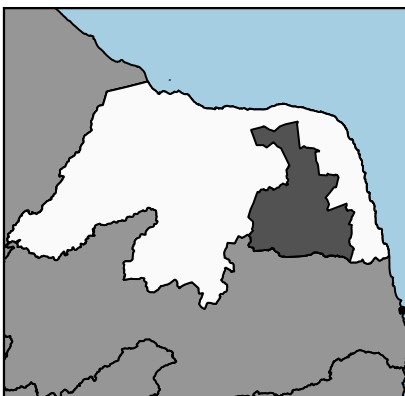

0 100 km

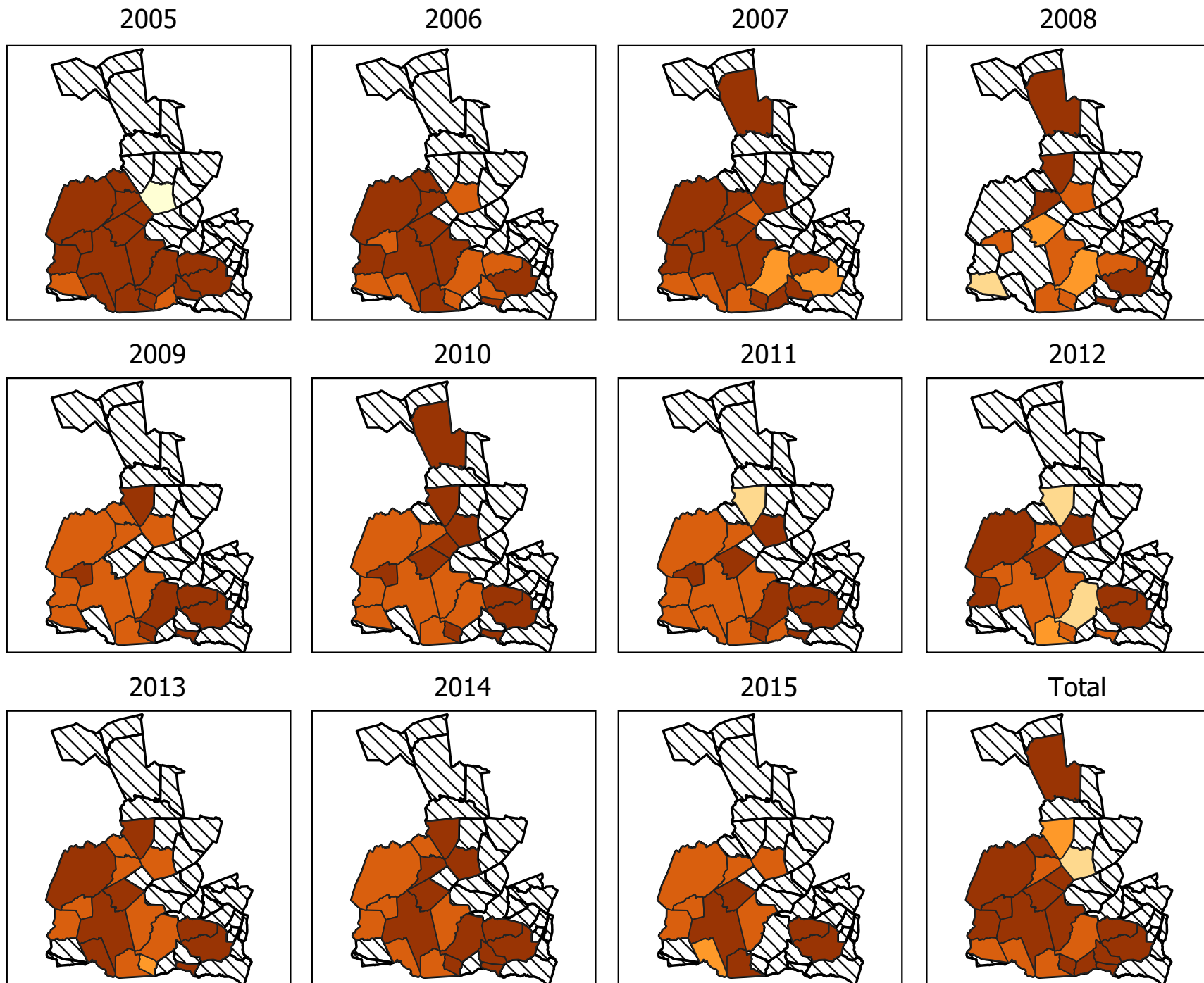

% HU Investigated

0 - 20 20 - 40 40 - 60 60 - 80 80 - 100 Municipalities not surveyed

0 25 50 75 100 km

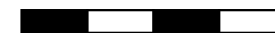

Supplement: S2 File — (PDF) [file pone.0287260.s002.pdf]

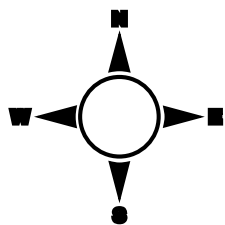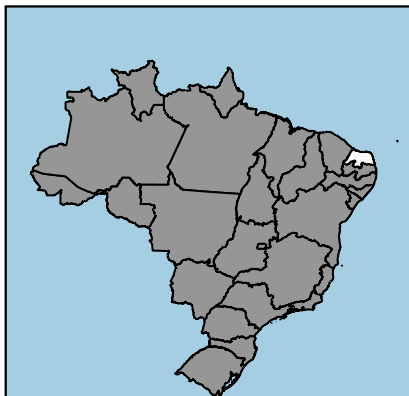

0 1,000 km

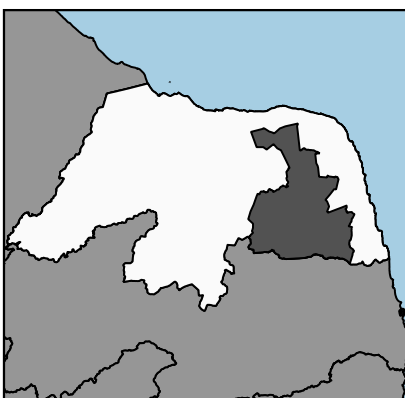

0 100 km

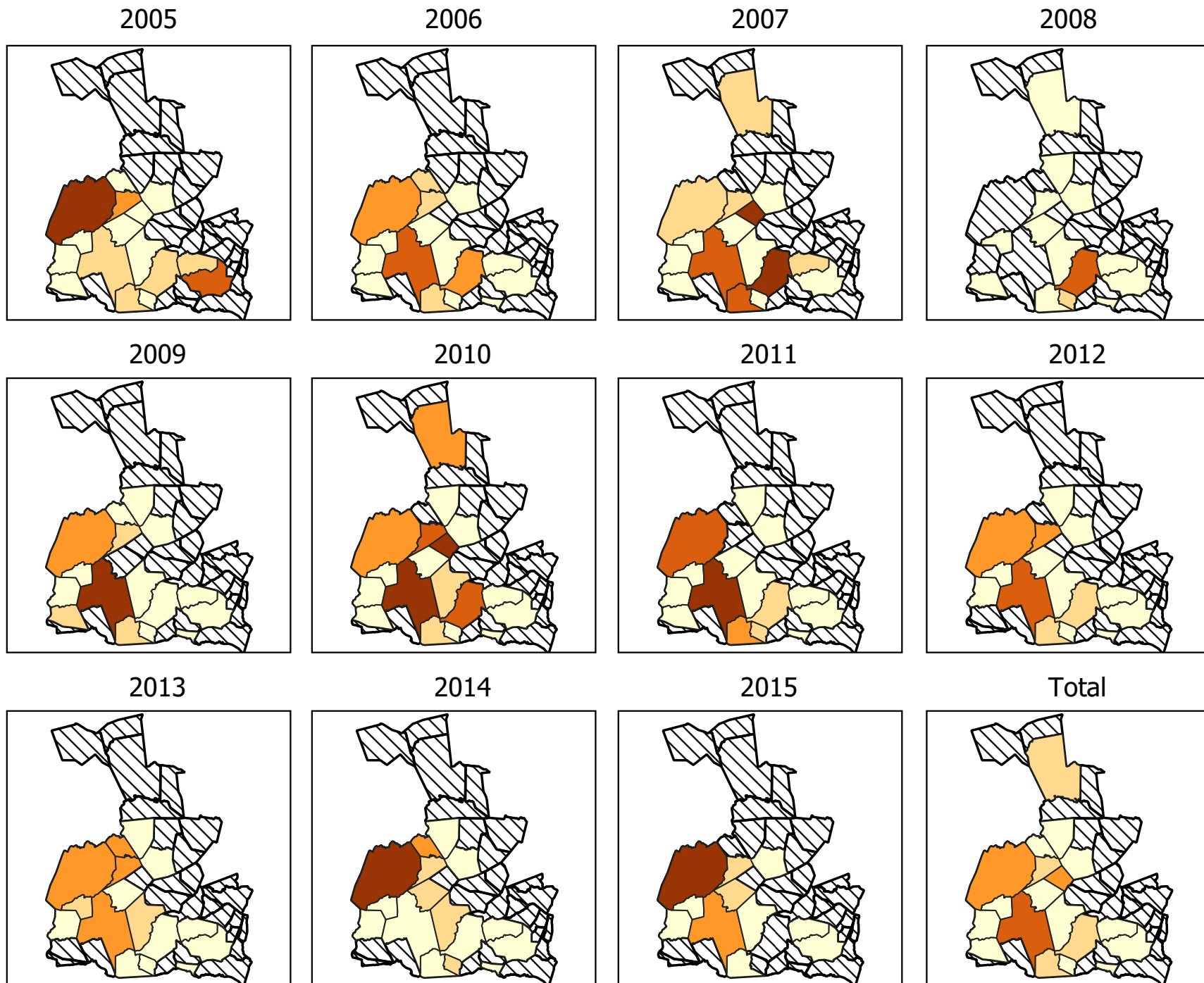

% HU Infested

0 - 2.5 2.5 - 5 5 - 7.5 7.5 - 10 10 - 100 Municipalities not surveyed

0 25 50 75 100 km

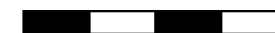

Supplement: S3 File — (PDF) [file pone.0287260.s003.pdf]

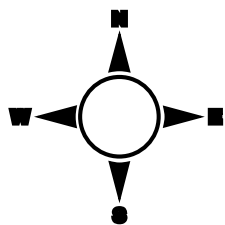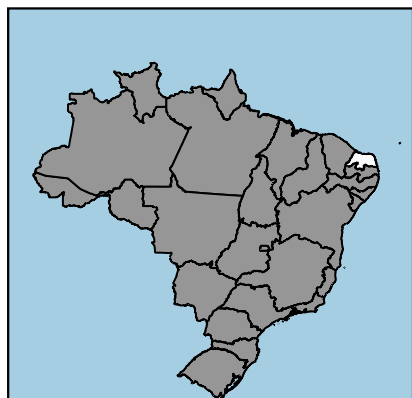

0 1,000 km

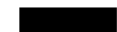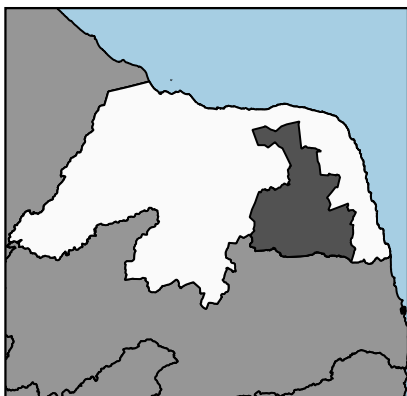

0 100 km

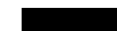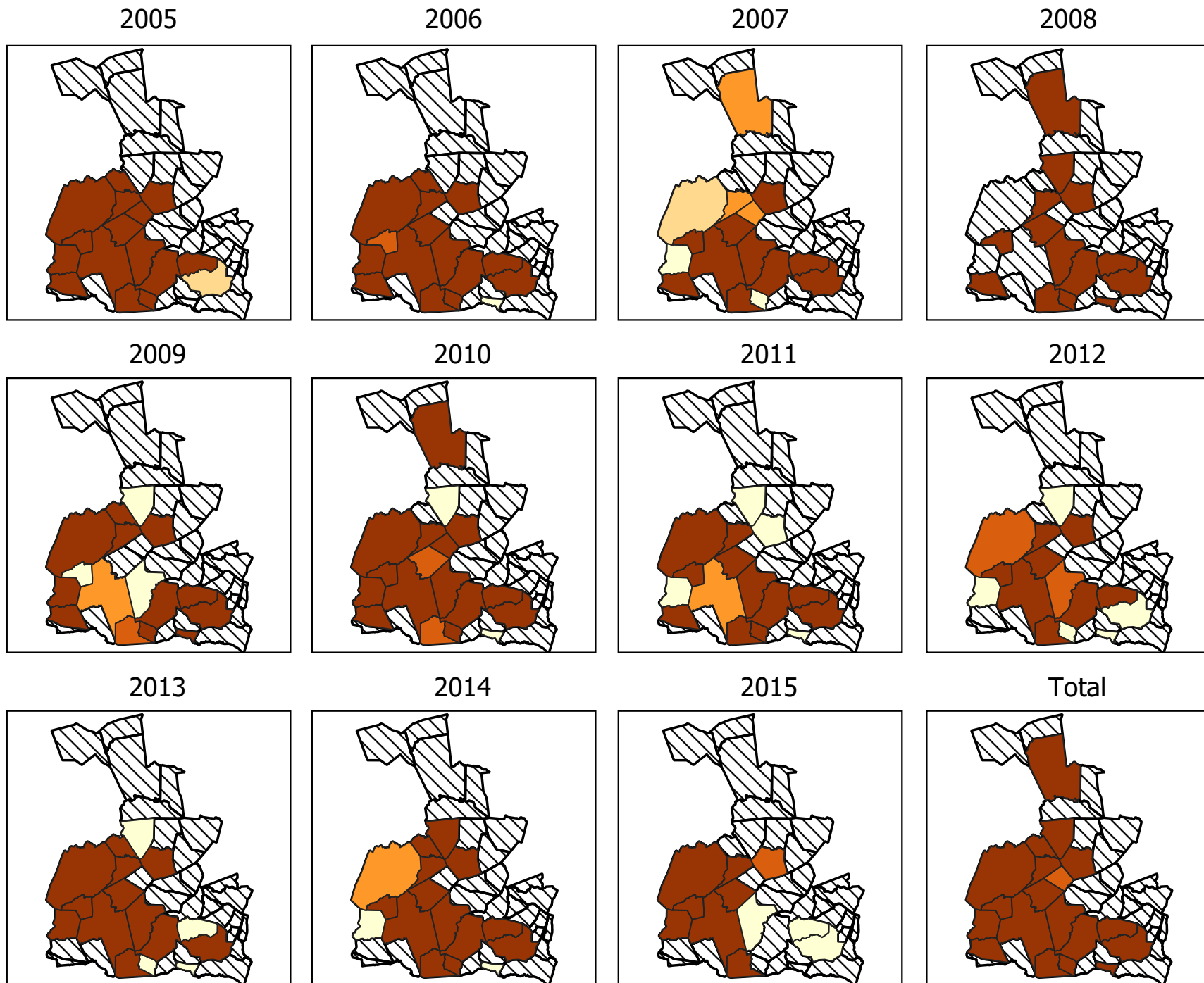

% HU Sprayed

0 - 40 40 - 55 55 - 70 70 - 85 85 - 100 Municipalities not surveyed

0 25 50 75 100 km

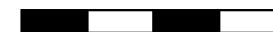

Supplement: S4 File — (PDF) [file pone.0287260.s004.pdf]

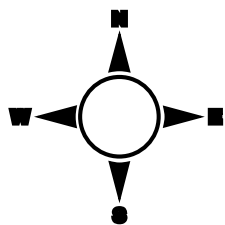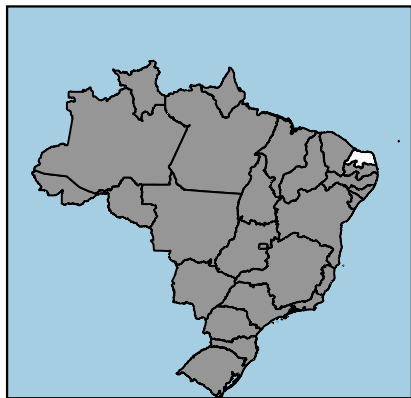

0 1,000 km

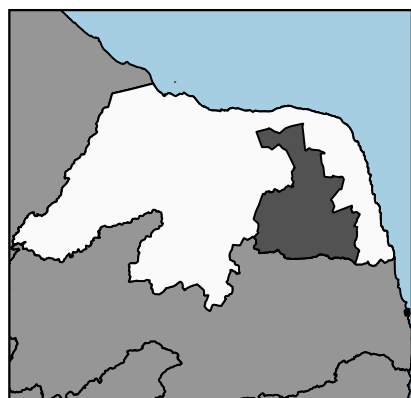

0 100 km

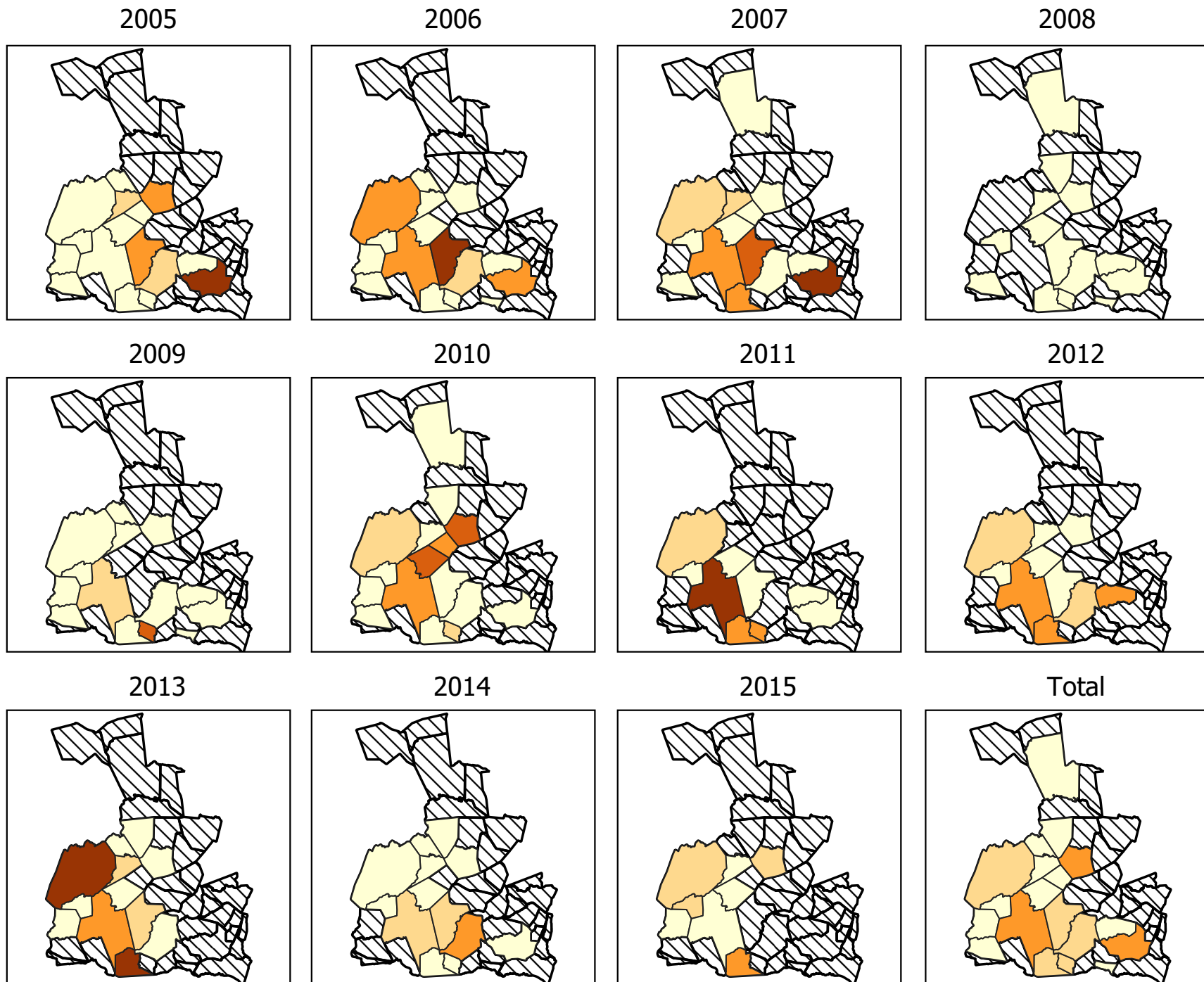

% HU Colonized

0 - 20 20 - 40 40 - 60 60 - 80 80 - 100 Municipalities not surveyed

0 25 50 75 100 km

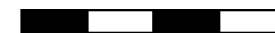

Supplement: S5 File — (PDF) [file pone.0287260.s005.pdf]
