# Supplementary material for: Temporal assessment of entomological surveillance of Trypanosoma cruzi vectors in an endemic area of northeastern Brazil
Source: PLoS One. 2023 Jun 15;18(6):e0287260. doi: 10.1371/journal.pone.0287260 (PMC10270571; doi:10.1371/journal.pone.0287260)
Supplement: Attachment — Submitted filename: Revision, PLOS ONE, Manuscript Number PONE-D-22-24351.docx [file pone.0287260.s006.docx]

The presented article describes the impact of triatomine surveillance and control measures between 2005 and 2015 in an endemic area for Chagas disease in the state of Rio Grande do Norte, Brazil.

This is an interesting approach and brings relevant information about vector control of triatomines in Agreste mesoregion, endemic area of Chagas disease, of the Rio Grande do Norte and describes changes in entomological surveillance metrics, used to monitor *T. cruzi* vectors in Brazil.

The methods are adequate in part to meet the proposed objectives, and the results and discussion are well described, needing some review and organization of the presented ideas. The conclusion section must be reviewed to align to presented objectives.

The manuscript has merits for be published in PLOS ONE, however, a few changes and corrections must be made before its acceptance.

**General Comments**

**1 –** Consider using the nomenclature “*T.cruzi*-like” instead of “*T.cruzi*” when referring to the diagnosis observed in the parasitological examination. Through the fresh parasitological examination, it is not possible to specifically distinguish *T. cruzi* from other species of *the Trypanosoma* genus, or even other flagellated protozoa of the Trypanosomatidae Family. Only molecular diagnosis can differentiate the species of the genus, and sometimes with a great difficulty.

**2 –** Consider review the objective: “Therefore, this study evaluated the impact of triatomine surveillance and control measures between 2005 and 2015 in an endemic area for ChD in the state of Rio Grande do Norte, Brazil.”

Here some arguments: your study evaluated the impact of triatomine surveillance on/over what? I mean, your objective was to describe the presented data or was to evaluate the impact over some specific determinant over the time? Please, consider reviewing the objectives to guide you along the Results, Discussion and Conclusion section. Also, these sections need to be better align with your objectives.

**3 –** Review the taxonomic rules of the scientific nomenclature. Review the abbreviations of *the Triatoma* genus and other genera along with all of the text.

**Methods**

**1 –** Describe your study design. Prospective, retrospective? Align the study methods to your scientific questions and main reviewed objectives.

Statistical Analysis

**2 –** Describe the software or packages used to analyze and geoprocessing the data.

**3 –** Do you describe β coefficient in the Results section, but don’t describe this analysis in the Methods section.

**4 –** Consider amplifying the impact of your results. For example, what is the impact of the data presented for Brazil and for the world?

**Results**

**1 –** In the first paragraph of the results, between lines 133 and 137, it describes information regarding the method session. Consider relocating the information to the correct section.

**2 –**

**Discussion**

**1 –** Consider reviewing all this section, better organize the ideas to maintain the same sequence of information described at the results section, and align to your objectives.

**Conclusions**

**1 –** Consider review this section to align with the proposed objective.

References

**1 –** Consider review, some references do not present the DOI, some are not adequately formatted.

**Specific Comments**

**Line 6** – “Active entomological” – these data also include captured triatomines reported by passive surveillance through PITs? (Triatomine Information Post).

**Line 25** – First taxonomic description of *Trypanosoma cruzi.* Consider include the complete description of the taxa *- Trypanosoma cruzi* (Chagas, 1909) – as presented for the triatomine names. Also, to the other taxonomic entities.

**Line 45** – Consider reviewing the argument presented as evidence of active transmission of T. cruzi. A study (https://pubmed.ncbi.nlm.nih.gov/21249297) showed that about 3.7% of mothers were infected with *T. cruzi* and 2.5% of their newborn children acquired the parasite congenitally. (mother and child). Other values indicate even higher numbers “Argentina (6%), in 292 of 7,086 in Bolivia (4.1%) and in 115 of 2,691 in Paraguay (4.3%)” (https://doi.org/10.1590/0074-02760140405). The data presented in your manuscript informs “...1 out of 1,750 samples analyzed from the state of RN was positive for anti-T. crossed antibodies…”. This indicates a prevalence of 0.058%, much lower than what was observed in transmission without the presence of the vector. Despite of this, these are epidemiologically distinct scenarios, so my suggestion to the authors is that they consider better developing the presented rationale of this paragraph.

**Line 50-52** – Since triatomine species have already been cited, consider writing the cited species in abbreviated form. Additionally, the full name of the species *P. megistus* has the wrong descriptor (Neiva and Pinto, 1923).

**Line 71** – instead of “better control with ChD” consider “better control of ChD transmission.”

**Line 100** – Please, consider better describing “exhaustively.”

**Line 105** – Consider *T.cruzi*-like when referring to parasitological test.

**Line 158-159** – Duplicated word - “index.”

**Line 165-167** – Once this triatomines species is already presented in the text, consider abbreviating their genus. *Triatoma pseudomaculata* as *T. pseudomaculata*.

Also, please observe *Triatoma Brasiliensis*.

**Lines 169-170** – Once these triatomine species are already presented in the text, consider abbreviating their genus.

**Lines 187-190** – Consider review the sentence: “The risk of transmission of *T. cruzi* persists due to the existence of autochthonous triatomine species with colonization potential and the persistence of residual foci of *Triatoma infestans* in both sylvatic [39] and domestic environments [40,41].” Here some arguments: This specie was not part of your sample, there are no longer colonies of this specie in Brazil, *Triatoma infestans* in sylvatic environments does not occur in Brazil.

**Lines 198** – Consider review the sentence: “The infested areas without residual spraying become a favorable habitat for the proliferation of triatomines, with abundant vertebrate hosts for blood-feeding because of the availability of humans and various species of domestic and peridomestic animals, in addition to resting sites and hiding places in the HU. Thus, spraying the adjacent surrounding areas prevents large-scale spatial displacement from infested areas of triatomines in search of bloodmeals.”

**Line 247-249** – Consider review the sentence: “The main objective of ChD control programs is to eradicate domestic colonies of triatomines and maintain continuous surveillance against new-colonization” by “The main objective of ChD vector control programs is to eradicate domestic colonies of triatomines and maintain continuous surveillance *of T. cruzi* vectors.”

**Line 253** – Consider review the sentence: “In low-risk municipalities” by “In municipalities classified as low-risk for *T. cruzi* transmission”

**Line 277** – Consider review the sentence: “have been reported” by “have been reported previously.”

**Line 288** – Consider review the sentence: “but a specificity of 100% for

*T. cruzi.”* Only molecular methods can differentiate *T. cruzi* from others Trypanosomatidae (Family). May I ask you, which were the taxonomic markers do you used to differentiate *T. cruzi* from *T. donovanni*, at parasitological examination, for example?

**Figures**

**Figure 1.** The presented figure needs to be intensively reviewed. It’s not necessary to keep the author of the figure at the figure. Two legends of color degrade are presented in the figure, but it’s not clear to which map it belongs, please review the figure. Consider insert legends (A, B and C) to each tree maps presented at the Figure 1. Also, better describe this information at Figure 1 description.
