# Supplementary material for: Temporal assessment of entomological surveillance of Trypanosoma cruzi vectors in an endemic area of northeastern Brazil
Source: PLoS One. 2023 Jun 15;18(6):e0287260. doi: 10.1371/journal.pone.0287260 (PMC10270571; doi:10.1371/journal.pone.0287260)
Supplement: Attachment — Submitted filename: Response to Reviewers PLOS ONE.docx [file pone.0287260.s008.docx]

The presented article describes the impact of triatomine surveillance and control measures between 2005 and 2015 in an endemic area for Chagas disease in the state of Rio Grande do Norte, Brazil.

This is an interesting approach and brings relevant information about vector control of triatomines in Agreste mesoregion, endemic area of Chagas disease, of the Rio Grande do Norte and describes changes in entomological surveillance metrics, used to monitor *T. cruzi* vectors in Brazil.

The methods are adequate in part to meet the proposed objectives, and the results and discussion are well described, needing some review and organization of the presented ideas. The conclusion section must be reviewed to align to presented objectives.

The manuscript has merits for be published in PLoS ONE, however, a few changes and corrections must be made before its acceptance.

**General Comments**

**1 –** Consider using the nomenclature “*T.cruzi*-like” instead of “*T.cruzi*” when referring to the diagnosis observed in the parasitological examination. Through the fresh parasitological examination, it is not possible to specifically distinguish *T. cruzi* from other species of *the Trypanosoma* genus, or even other flagellated protozoa of the Trypanosomatidae Family. Only molecular diagnosis can differentiate the species of the genus, and sometimes with a great difficulty.

**1 - Answer**: The term the nomenclature “*T.cruzi*-like” was used as suggested. Please see in the text of the manuscript highlighted yellow.

**2 –** Consider review the objective: “Therefore, this study evaluated the impact of triatomine surveillance and control measures between 2005 and 2015 in an endemic area for ChD in the state of Rio Grande do Norte, Brazil.”

Here some arguments: your study evaluated the impact of triatomine surveillance on/over what? I mean, your objective was to describe the presented data or was to evaluate the impact over some specific determinant over the time? Please, consider reviewing the objectives to guide you along the Results, Discussion and Conclusion section. Also, these sections need to be better align with your objectives.

**2 - Answer**: The objectives have been revised and edited as suggested. . Please see in the text of the manuscript highlighted yellow. The new objectives has been aligned with the results, discussion and conclusion sections.

**3 –** Review the taxonomic rules of the scientific nomenclature. Review the abbreviations of *the Triatoma* genus and other genera along with all of the text.

**3 - Answer**: The taxonomic rules of scientific nomenclature have been revised, particulary the abbreviations of the genus *Triatoma* and other genera. Please see in the text of the manuscript highlighted yellow.

**Methods**

**1 –** Describe your study design. Prospective, retrospective? Align the study methods to your scientific questions and main reviewed objectives.

**1 - Answer**: The study design was described and aligned with the methods, its scientific questions and the main objectives reviewed as suggested. Please see in the text of the manuscript highlighted yellow.

**Statistical Analysis**

**2 –** Describe the software or packages used to analyze and geoprocessing the data.

**2 - Answer**: The software used to analyze and geoprocess the data was described in the methods section. Please see in the text of the manuscript highlighted yellow.

**3 –** Do you describe β coefficient in the Results section, but don’t describe this analysis in the Methods section.

**3 - Answer**: The β coefficient was described in the methods section. Please see in the text of the manuscript highlighted yellow.

**4 –** Consider amplifying the impact of your results. For example, what is the impact of the data presented for Brazil and for the world?

**4 - Answer**: The reviewer`s suggestion was accepted. Please see in the text of the manuscript highlighted yellow.

**Results**

**1 –** In the first paragraph of the results, between lines 133 and 137, it describes information regarding the method session. Consider relocating the information to the correct section.

**1 - Answer:** In the first paragraph the results that described information regarding the method session were relocated in the correct section as suggested. Please see in the text of the manuscript highlighted yellow.

**Discussion**

**1 –** Consider reviewing all this section, better organize the ideas to maintain the same sequence of information described at the results section, and align to your objectives.

**1 - Answer**: The reviewer`s suggestion was accepted and the following of information and ideas presented in the objective were aligned with the results and discussion sections as suggested. Please see in the text of the manuscript highlighted yellow.

**Conclusions**

**1 –** Consider review this section to align with the proposed objective.

**1 - Answer**: This section was aligned with the proposed new objective as suggested. Please see in the text of the manuscript highlighted yellow.

**References**

**1 –** Consider review, some references do not present the DOI, some are not adequately formatted.

**1 - Answer**: References have been reviewed and formatted properly as suggested.

**Specific Comments**

**Line 6** – “Active entomological” – these data also include captured triatomines reported by passive surveillance through PITs? (Triatomine Information Post).

**Answer**: No, these data refer only to Triatomines captured by endemic agents in households.

**Line 25** – First taxonomic description of *Trypanosoma cruzi.* Consider include the complete description of the taxa *- Trypanosoma cruzi* (Chagas, 1909) – as presented for the triatomine names. Also, to the other taxonomic entities.

**Answer**: The first taxonomic description of *Trypanosoma cruzi* and other taxonomic entities were included as suggested. Please see in the text of the manuscript highlighted yellow.

**Line 45** – Consider reviewing the argument presented as evidence of active transmission of *T. cruzi*. A study (https://pubmed.ncbi.nlm.nih.gov/21249297) showed that about 3.7% of mothers were infected with *T. cruzi* and 2.5% of their newborn children acquired the parasite congenitally. (mother and child). Other values indicate even higher numbers “Argentina (6%), in 292 of 7,086 in Bolivia (4.1%) and in 115 of 2,691 in Paraguay (4.3%)” (https://doi.org/10.1590/0074-02760140405). The data presented in your manuscript informs “...1 out of 1,750 samples analyzed from the state of RN was positive for anti-T. crossed antibodies…”. This indicates a prevalence of 0.058%, much lower than what was observed in transmission without the presence of the vector. Despite of this, these are epidemiologically distinct scenarios, so my suggestion to the authors is that they consider better developing the presented rationale of this paragraph.

**Answer**: The argument presented as evidence of active transmission of *T. cruzi* in the studied region, regarding seropositivity in children, was presented in another order of importance in the text as suggested. Please see in the text of the manuscript highlighted yellow.

**Line 50-52** – Since triatomine species have already been cited, consider writing the cited species in abbreviated form. Additionally, the full name of the species *P. megistus* has the wrong descriptor (Neiva and Pinto, 1923).

**Answer**: Triatomine species were abbreviated after being cited. In addition, the full name of the *P. megistus* species has been corrected. Please see in the text of the manuscript highlighted yellow.

**Line 71** – instead of “better control with ChD” consider “better control of ChD transmission.”

**Answer**: The reviewer`s suggestion was accepted. Please see in the text of the manuscript highlighted yellow.

**Line 100** – Please, consider better describing “exhaustively.”

**Answer**: The word “exhaustively” has been removed from the text. Please see in the text of the manuscript highlighted yellow.

**Line 105** – Consider *T. cruzi*-like when referring to parasitological test.

**Answer**: The reviewer`s suggestion was accepted. Please see in the text of the manuscript highlighted yellow.

**Line 158-159** – Duplicated word - “index.”

**Answer**: Duplicated word - “index” has been removed from the text. Please see in the text of the manuscript highlighted yellow.

**Line 165-167** – Once this triatomines species is already presented in the text, consider abbreviating their genus. *Triatoma pseudomaculata* as *T. pseudomaculata*.

Also, please observe *Triatoma Brasiliensis*.

**Answer**: The genus was later abbreviated and *Triatoma Brasiliensis”* was corrected*.*

**Lines 169-170** – Once these triatomine species are already presented in the text, consider abbreviating their genus.

**Answer**: Your suggestion was accepted. Please see in the text of the manuscript highlighted yellow.

**Lines 187-190** – Consider review the sentence: “The risk of transmission of *T. cruzi* persists due to the existence of autochthonous triatomine species with colonization potential and the persistence of residual foci of *Triatoma infestans* in both sylvatic [39] and domestic environments [40,41].” Here some arguments: This specie was not part of your sample, there are no longer colonies of this specie in Brazil, *Triatoma infestans* in sylvatic environments does not occur in Brazil.

**Answer**: The sentence regarding *Triatoma infestans* has been removed as suggested. Please see in the text of the manuscript highlighted yellow.

**Lines 198** – Consider review the sentence: “The infested areas without residual spraying become a favorable habitat for the proliferation of triatomines, with abundant vertebrate hosts for blood-feeding because of the availability of humans and various species of domestic and peridomestic animals, in addition to resting sites and hiding places in the HU. Thus, spraying the adjacent surrounding areas prevents large-scale spatial displacement from infested areas of triatomines in search of bloodmeals.”

**Answer**: The sentence has been revised and rewritten more linearly. Please see in the text of the manuscript highlighted yellow.

**Line 247-249** – Consider review the sentence: “The main objective of ChD control programs is to eradicate domestic colonies of triatomines and maintain continuous surveillance against new-colonization” by “The main objective of ChD vector control programs is to eradicate domestic colonies of triatomines and maintain continuous surveillance *of T. cruzi* vectors.”

**Answer**: Your suggestion was accepted. Please see in the text of the manuscript highlighted yellow.

**Line 253** – Consider review the sentence: “In low-risk municipalities” by “In municipalities classified as low-risk for *T. cruzi* transmission”

**Answer**: The sentence was revised as suggested. Please see in the text of the manuscript highlighted yellow.

**Line 277** – Consider review the sentence: “have been reported” by “have been reported previously.”

**Answer**: The sentence was revised as suggested. Please see in the text of the manuscript highlighted yellow.

**Line 288** – Consider review the sentence: “but a specificity of 100% for*T. cruzi.”* Only molecular methods can differentiate *T. cruzi* from others Trypanosomatidae (Family). May I ask you, which were the taxonomic markers do you used to differentiate *T. cruzi* from *T. donovanni*, at parasitological examination, for example?

Answer: The sentence was revised as suggested. Please see in the text of the manuscript highlighted yellow.

**Figures**

**Figure 1.** The presented figure needs to be intensively reviewed. It’s not necessary to keep the author of the figure at the figure. Two legends of color degrade are presented in the figure, but it’s not clear to which map it belongs, please review the figure. Consider insert legends (A, B and C) to each tree maps presented at the Figure 1. Also, better describe this information at Figure 1 description.

**Answer**: Figure 1 was reviewed as suggested. Please see the new Figure 1.
